# Evaluating the association between *DNM1L* variants and Parkinson's disease in the Chinese population

**DOI:** 10.3389/fneur.2023.1133449

**Published:** 2023-02-24

**Authors:** Jiabin Liu, Juanjuan Huang, Yuwen Zhao, Hongxu Pan, Yige Wang, Zhenhua Liu, Qian Xu, Qiying Sun, Jieqiong Tan, Xinxiang Yan, Jinchen Li, Beisha Tang, Jifeng Guo

**Affiliations:** ^1^Department of Neurology, Xiangya Hospital, Central South University, Changsha, Hunan, China; ^2^National Clinical Research Center for Geriatric Disorders, Xiangya Hospital, Central South University, Changsha, Hunan, China; ^3^Center for Medical Genetics & Hunan Key Laboratory of Medical Genetics, School of Life Sciences, Central South University, Changsha, Hunan, China; ^4^Key Laboratory of Hunan Province in Neurodegenerative Disorders, Central South University, Changsha, Hunan, China; ^5^Hunan International Scientific and Technological Cooperation Base of Neurodegenerative and Neurogenetic Diseases, Changsha, China; ^6^Engineering Research Center of Hunan Province in Cognitive Impairment Disorders, Central South University, Changsha, China

**Keywords:** Parkinson's disease, *DNM1L*, DRP1, mitochondria, rare variants, common variants

## Abstract

**Introduction:**

Parkinson's disease (PD) is a progressive movement disorder caused by a loss of dopaminergic neurons. Previous studies have highlighted the importance of mitochondria dynamics in the pathogenesis of PD. Dynamin-1-like (*DNM1L*) is a gene that encodes dynamin-related protein 1 (DRP1), a GTPase essential for proper mitochondria fission. In the present study, we evaluated the relationship between *DNM1L* variants and PD in the Chinese population.

**Methods:**

A total of 3,879 patients with PD and 2,931 healthy controls were recruited and burden genetic analysis combined with high-throughput sequencing was applied.

**Results:**

We identified 23 rare variants in the coding region of *DNM1L*, while no difference in variant burden was shown between the cases and controls. We also identified 201 common variants in the coding and flanking regions and found two significant SNPs, namely, rs10844308 and rs143794289 [odds ratio (OR) = 1.220 and 0.718, *p* = 0.025 and 0.036, respectively]. We also performed a meta-analysis to correlate the two SNPs with PD risk. However, none of the common variants was significant using logistic regression.

**Conclusion:**

Despite the critical role of DRP1, our study did not support the relationship between *DNM1L* variants and PD risk in the Chinese population.

## Introduction

Parkinson's disease (PD) is the second most common neurodegenerative disorder affecting 2%−3% of individuals aged above 65 years (1). The major manifestations of PD are motor deficits, including bradykinesia, resting tremor, rigidity, and postural instability, mainly resulting from the death of cells in the substantia nigra (2). As widely known, many PD-related genes are directly associated with mitochondria, a highly dynamic organelle undergoing continuous fission and fusion (3). The balance between fission and fusion of mitochondria is fundamental for maintaining mitochondrial morphology, size, position, and transport within cells (4). In humans, the dynamin-1-like (DNM1L) gene encodes dynamin-related protein 1 (DRP1)—a multidomain GTPase required for mitochondrial fission. Under various cellular stimuli, DRP1 would translocate from the cytosol to the mitochondria to initiate the division of mitochondrial membranes through GTP hydrolysis (5). Dysregulation of DRP1 can trigger mitochondrial fragmentation and subsequently mitochondrial depolarization.

Given the close relationship between mitochondria and DRP1, *DNM1L* variants are likely related to many mitochondria-related diseases. In recent years, with the development of sequencing, some rare neurological diseases were found to be caused by *DNM1L* mutations. Previous studies reported that *DNM1L* variants are related to encephalopathy with mitochondria fission defects. The patients might have severe psychomotor retardation, dystonia, and epilepsy, indicating an essential role of *DNM1L* in the nervous system (6–8). Recently, the dysfunction of DRP1 has been identified in numerous PD models (9, 10). Notably, some studies found that the expression level of DRP1 was significantly reduced in the peripheral blood of patients with PD (11). However, there has been little discussion about the relationship between *DNM1L* variants and the risk of PD. Therefore, this study comprehensively evaluated the association between rare variants of *DNM1L* in large PD cohorts. We performed a genetic analysis on 1,917 patients with familial or sporadic early-onset PD (FPD/sEOPD) and 1,652 healthy controls as well as 1,962 patients with sporadic late-onset PD (sLOPD) and 1,279 healthy controls from mainland China. To elucidate the correlation between the variants of *DNM1L* in PD risk, burden genetic analysis combined with high-throughput sequencing was applied.

## Materials and methods

### Participants

Subjects were enrolled in the large cohort of Parkinson's Disease and Movement Disorders Multicenter Database and Collaborative Network in China (PD-MDCNC, http://www.pd-mdcnc.com/), as described in a previous study (12). Patients with PD in this cohort were diagnosed by experienced neurologists according to the UK Parkinson's Disease Scoeity (PDS) Brain Bank Criteria (13) or Movement Disorder Society Clinical Diagnostic Criteria (14). Meanwhile, neurological disease-free controls were recruited, with matched ethnicity *as a reference*. As indicated in our earlier study, we excluded individuals with pathogenic/likely pathogenic mutations of 23 PD pathogenic genes from the EOPD and FPD cohorts. These patients were divided into two separate cohorts, which were named according to the sequencing method as follows: (1) *the* whole-exome sequencing (WES) cohort, containing 1,917 familial or sporadic early-onset PD from Mainland China (mean age, 52.22 ± 9.03 years; *women*, 45.4%) and 1,652 race-matched healthy controls (mean age, 62.03 ± 12.59 years; *women*, 51.9%), and (2) *the* whole-genome sequencing (WGS) cohort, containing 1,962 sporadic late-onset PD from Mainland China (mean age, 66.76 ± 7.078 years; *women*, 49.8%) and 1,652 race-matched healthy controls (mean age, 62.32 ± 7.109 years; *women*, 52.1%). The WES cohort consisted of 477 familial PD probands (327 AD probands and 150 AR probands) and 1,440 patients with early-onset PD (age of onset was not more than 50 years). The WGS cohort contained patients with late-onset sporadic PD with mean age onset of 61.88 ± 6.927 years. Patients in both cohorts carrying pathogenic mutations in high-confidence PD-causing genes were excluded from the analysis. Followed by informed consent, the basic demographic data, peripheral blooding samples, and clinical features of all participants were collected. We collected total genomic DNA from peripheral blooding samples using standard procedures. The respective ethics committees of Xiangya Hospital of Central South University approved the abovementioned protocol.

### Sequencing and quality control

As shown in our previous study (15), the WES cohort used SureSelect Human All Exon Kit V6 (Agilent) to capture the whole-exome DNA, prepared the sample library, and then used Illumina HiSeq 10× for pair-end 2 × 150 bp sequencing. The average sequencing depth was 123×, achieving a coverage of at least 10× for 99.32% of the target region. WGS was performed using the Illumina Nova Sequencing platform in a pair-end 2 × 150 bp mode, and the average depth of coverage was about 12×. Sequencing data of both groups were processed and analyzed with the BWA-GATK-ANNOVAR pipeline (16, 17). Quality control was conducted as described in our previous study (15). Samples would be removed if they had sex discrepancies, abnormal heterozygosity (>3 SD), pathogenic or likely pathogenic variants of PD-related genes, or unusual relatedness (descent > 0.15). In addition, we performed the principal component analysis (PCA) using PLINK v1.90 to assess potential population structure stratification. In subsequent analyses for the WGS cohort, gender, age, and the first five principal components of population stratification were used as covariates, whereas for the WES cohort (the control group included elderly people without neurological diseases), sex and the first five principal components for population stratification were used (18).

### Variant definition and association analysis

In both two cohorts, we targeted the variants in the coding region of the *DNM1L* gene (NM_001278466). The variants with a missing rate of >5% and deviations from Hardy–Weinberg equilibrium in controls (*p* < 0.05) were removed using PLINK v1.90. We annotated the gene regions (hg19 RefSeq), amino acid changes, and allele frequency of each variant in the Genomic Aggregation Database (gnomAD) and Exon Aggregation Consortium (ExAC) by ANNOVAR. Next, the functional impact of each nonsynonymous variant was predicted by ReVe (threshold, 0.7).

We categorized the variants according to the minor allele frequency (MAF) as common variants (MAF > 0.01) and rare variants (MAF < 0.01). Furthermore, we re-extracted the rare variants with MAF below 0.001 and performed gene analysis of rare variants with MAF below 0.01 and 0.001, respectively. According to predicted functions, all the rare nonsynonymous variants were classified into three variant groups: missense, potentially damaging missense (Dmis, ReVe score > 0.7), and loss of function variants (LoF stop gain/loss, frameshift, and splice site), and the sum of Dmis and LoF. After adjusting the abovementioned covariates in two cohorts, sequence kernel association test-optimal (SKAT-O) was applied to each cohort to assess the combined effect of rare variants and each variants group. Fisher's exact test was also done for common variants to validate the significant relationship between the common variants of *DNM1L* and PD. Moreover, logistic regression analysis based on an allele model was also performed by PLINK v1.90, and a *p*-value of < 0.05 was considered suggestive significant.

### Meta-analysis

To confirm the involvement of *DNM1L* variants in PD susceptibility, a meta-analysis combining public studies and our case–control study was conducted. In addition to our data, summary data from the PD variant browser were included in the meta-analysis, which comprised four studies: PD Genome Project, International Parkinson's Disease Genomic Consortium (IPDGC) Exomes, IPDGC Resequencing Project, and UK Biobank (19). Unlike our cohorts, these cohorts mainly contain European populations, which can increase the power to detect the association between *DNM1L* variants and PD. As no rare variants of *DNM1L* found in our cohort were seen in included public data, we only validated the significant association between significant common variants of *DNM1L* and PD in meta-analysis. We used the Hardy-Weinberg equilibrium model to estimate the SNPs in all cohorts and then excluded the variants with deviations in controls (*p* < 0.05). To assess the strength of the association between *DNM1L* variants and PD risk, a pooled OR and 95% confidence intervals (CIs) were calculated under five different models (allele, dominant, recessive, heterozygote, and homozygote model). The Cochrane *Q*-test and *I*^2^ statistics were used to assess study heterogeneity and a significant *Q*-test (*p* < 0.1 or *I*^2^ > 50%) indicated heterogeneity. Fixed- or random-effects models were selected based on the presence or absence of heterogeneity. A *Z*-test determined the significance of the pooled ORs. We used the FDR method to correct *p*-values for multiple comparisons for the variant association analysis. A *p*-value of < 0.05 was considered statistically significant. Moreover, the *p*-values of Egger's and Begg's tests were calculated to estimate the publication bias. All analyses were performed using the R package “meta.”

## Results

### *DNM1L* variants identification

The basic demographic characteristics of the two cohorts are summarized in Supplementary Table S1. In the WES cohort, a total of 1,917 familial PD probands or sporadic early-onset cases and 1,652 controls were included. The WGS cohort included 1,962 sporadic late-onset PD (sLOPD) patients and 1,279 healthy controls. After variant filtering and classification, we identified 224 variants in the coding region of *DNM1L*, including 23 rare nonsynonymous variants and 201 common variants.

**Figure 1:**
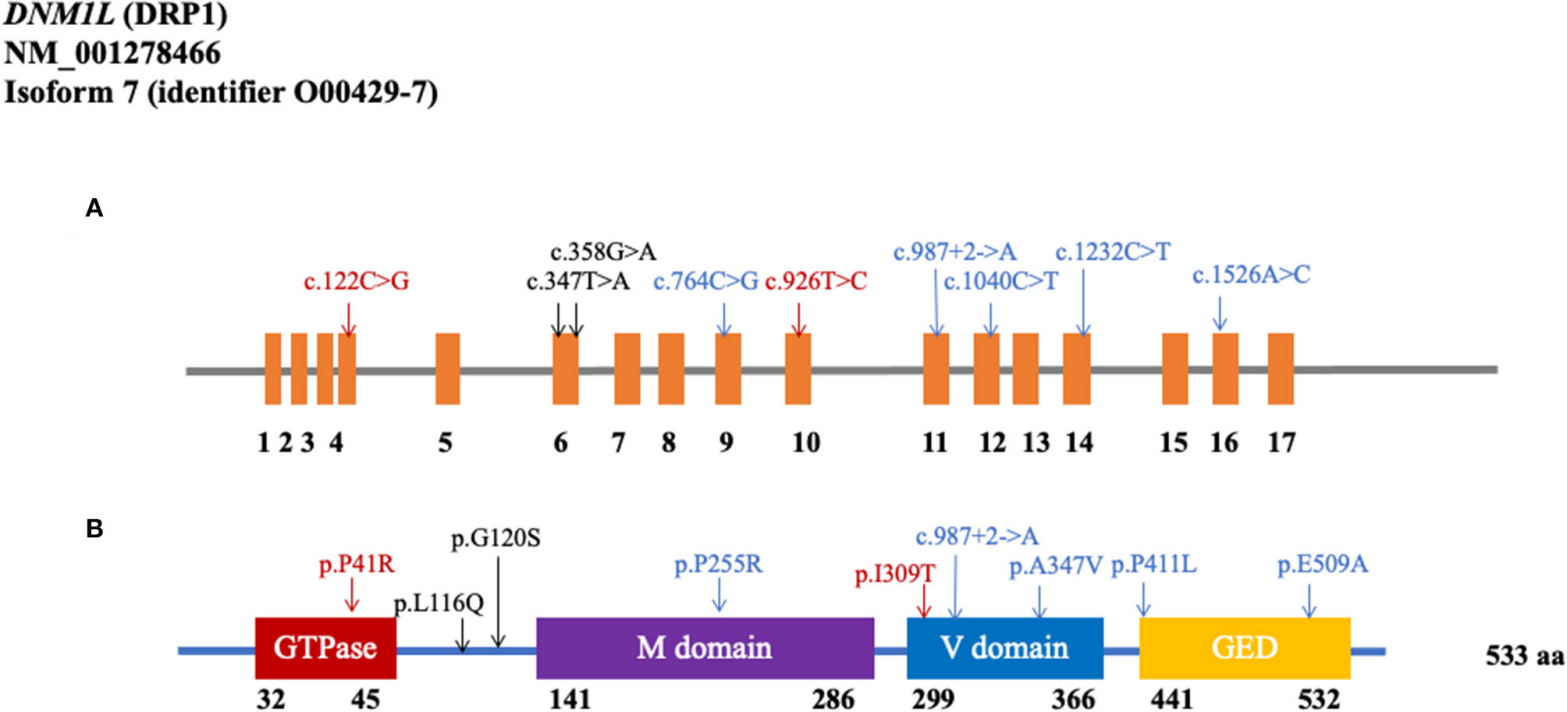
Rare variants in the coding region of DNM1L. **(A)** Schematics of rare nonsynonymous variants in the coding region of *DNM1L*. **(B)** Location of potential damaging rare variants in DRP1 protein (NM_001278466). DRP1 contains the GTPase domain, middle (M) domain, variable (V) domain, and GTPase effector domain (GED). The position of each domain was provided by https://www.uniprot.org/uniprot/O00429 (Black: WES cohort; Blue: WGS cohort; Red: shared by two cohorts).

As shown in Figure 1 and Table 1, the MAF of the 23 rare variants was below 0.001. Of these 23 rare variants, 21 of them were missense mutations, and 12 were predicted to be damaging. Of the 12 damaging missenses, five of them were only found in patients with PD (p.V213A, p.L116Q, p.G120S, p.P255R, and p.P411L). In addition, we found two shear mutations that may lead to loss of function (c.987+2_987+3insA and c.988-1_988insTCT). In detail, c.987+2_987+3insA was detected in one control from the WES cohort and in two patients from the WGS cohort, whereas c.988-1_988insTCT was detected in only one patient from the WGS cohort.

**Table 1:** Rare nonsynonymous variants of *DNM1L* identified in our cohort.

**Gene**	**Position (hg19)**	**Ref**	**Alt**	**NM number**	**AAChange**	**Consequence**	**gnomAD_ exome_ EAS^a^**	**gnomAD_ genome_ EAS^a^**	**ExAC_ EAS^a^**	**ReVe^b^**	**WES cohort**		**WGS cohort**	
											**Case (*****n*** = **1,917)**	**Control (*****n*** = **1,652)**	**Case (*****n*** = **1,962)**	**Control (*****n*** = **1,279)**
*DNM1L*	12:32854394	C	G	NM_012062	c.148C>G:p.L50V	Missense	0	–	0	0.629:T	0	1	0	0
*DNM1L*	12:32854472	C	T	NM_012062	c.226C>T:p.R76W	Missense	0	0	–	0.755:D	0	1	0	0
*DNM1L*	12:32854483	A	G	NM_001278466	c.32A>G:p.Q11R	Missense	–	–	–	0.641:T	0	1	0	0
*DNM1L*	12:32860338	C	G	NM_001278466	c.122C>G:p.P41R	Missense	0.0004	0.0006	0.0005	0.805:D	3	4	5	1
*DNM1L*	12:32863906	A	G	NM_012062	c.413A>G:p.N138S	Missense	0.0005	0	0.0003	0.333:T	0	1	1	2
*DNM1L*	12:32866197	C	A	NM_012062	c.511C>A:p.L171I	Missense	0.0001	–	0.0001	0.586:T	9	3	6	4
*DNM1L*	12:32871595	T	C	NM_012062	c.638T>C:p.V213A	Missense	–	–	–	0.934:D	1	0	0	0
*DNM1L*	12:32871666	G	A	NM_012062	c.709G>A:p.V237I	Missense	–	–	–	0.575:T	1	0	0	0
*DNM1L*	12:32875444	T	A	NM_001278466	c.347T>A:p.L116Q	Missense	–	–	–	0.988:D	1	0	0	0
*DNM1L*	12:32875455	G	A	NM_001278466	c.358G>A:p.G120S	Missense	–	–	–	0.891:D	1	0	0	0
*DNM1L*	12:32884804	C	G	NM_001278466	c.764C>G:p.P255R	Missense	–	–	–	0.957:D	0	0	1	0
*DNM1L*	12:32886737	T	C	NM_001278466	c.926T>C:p.I309T	Missense	0.0002	0.0006	0.0003	0.788:D	1	1	2	1
*DNM1L*	12:32890081	G	C	NM_001278466	c.973G>C:p.V325L	Missense	0.0002	–	0.0001	0.401:T	1	2	0	1
*DNM1L*	12:32890097	–	A	NM_001278466	c.987+2_987+3insA	Splicing	0	–	0	–	0	1	2	0
*DNM1L*	12:32890798	–	TCT	NM_001278466	c.988-1_988insTCT	Splicing	0.00005798	–	–	–	1	0	0	0
*DNM1L*	12:32890803	C	G	NM_001278466	c.992C>G:p.S331C	Missense	–	–	–	0.449:T	1	0	0	0
*DNM1L*	12:32890824	C	T	NM_001278466	c.1013C>T:p.A338V	Missense	0.00005798	–	0.0001	0.480:T	0	1	0	0
*DNM1L*	12:32890850	G	A	NM_001278466	c.1039G>A:p.A347T	Missense	0.00005798	0	0	0.757:D	0	1	0	0
*DNM1L*	12:32890851	C	T	NM_001278466	c.1040C>T:p.A347V	Missense	–	–	–	0.771:D	0	1	3	0
*DNM1L*	12:32893068	T	A	NM_001278466	c.1169T>A:p.L390Q	Missense	–	–	–	0.907:D	0	1	0	0
*DNM1L*	12:32893131	C	T	NM_001278466	c.1232C>T:p.P411L	Missense	–	–	–	0.764:D	0	0	1	0
*DNM1L*	12:32895611	T	G	NM_001278466	c.1474T>G:p.L492V	Missense	–	–	–	0.519:T	0	1	0	0
*DNM1L*	12:32895663	A	C	NM_001278466	c.1526A>C:p.E509A	Missense	0.00005798	0	0	0.809:D	0	0	0	1

a Variants minor allele frequencies from gnomAD_genome_EAS, gnomAD_exome_EAS, and ExAC_EAS.

b The score of prediction software:predictive results; D, damaging; T, tolerate.

### Association analysis results

Table 2 shows the results of the rare variants association test of *DNM1L*. To increase the power to detect association, we performed the burden analyses on WES and WGS cohorts stratified by the functional effects of variants. As a result, no relationship between PD and variants was observed in any variants groups (*p* > 0.05, Table 2).

**Table 2:** Analysis of *DNM1L* genes rare variant burden in Parkinson's disease.

**Variants group**	**WES cohort**						**WGS cohort**					
	**(a) MAF**<**1%**			**(b) MAF**<**0.1%**			**(c) MAF**<**1%**			**(d) MAF**<**0.1%**		
	**Cases (*****n*** = **1,917)**	**Controls (*****n*** = **1,652)**	^*^ ***p*** **-value**	**Cases (*****n*** = **1,917)**	**Controls (*****n*** = **1,652)**	***p*** **-value**	**Cases (*****n*** = **1,962)**	**Controls (*****n*** = **1,279)**	^*^ ***p*** **-value**	**Cases (*****n*** = **1,962)**	**Controls (*****n*** = **1,279)**	^*^ ***p*** **-value**
(1) Missense	19	19	0.745	19	19	0.745	19	10	0.799	19	10	0.799
(2) Dmis	7	9	0.716	7	9	0.716	12	3	0.234	12	3	0.234
(3) LoF	1	1	0.271	1	1	0.271	2	0	0.999	2	0	0.999
(4) LoF + Dmis	8	10	0.662	8	10	0.662	14	3	0.234	14	3	0.234

* p-value was calculated by Fisher's exact test/SKAT-O.

Dmis, damaging missense variants.

The bold value means the *p* value < 0.05, suggesting significance.

Among the 201 common variants, only two common variants (rs10844308 and rs143794289) in the WES cohort were significant in the Fisher's test. Also, rs10844308 was suggested to cause a 22% increase in the odds of PD (OR = 1.220, *p* = 0.036). On the contrary, rs143794289 was presented as a protective factor of PD since it caused a 28.2% decrease in the odds of PD (OR = 0.718, *p* = 0.025). However, the result of these two SNPs turned negative in the WGS cohort analysis. Logistic regression with PLINK also analyzed associations between common variants and PD. Age at enrollment, sex, and the first five principal components was used as adjustment covariates. Nevertheless, logistic regression did not demonstrate any suggestive result between *DNM1L* and PD (*p* > 0.05, Supplementary Tables S2, S3).

### Meta-analysis of *DNM1L* common variants and PD

As mentioned earlier, we found two significant common variants in the WES cohort—rs10844308 and rs143794289. These two SNPs were further included in the meta-analysis. For all included studies, genotypes of these two variants in controls were consistent with Hardy–Weinberg equilibrium (*p* > 0.05). With regard to rs10844308, data were obtained from six cohorts (WES, WGS, and four public cohorts), including 28,985 patients and 69,206 controls. In both the Chinese population and European populations, no genetic models showed evidence of heterogeneity (*p* for heterogeneity > 0.1, *I*^2^ < 50). Therefore, we applied a fixed-effects model for all the analyses of rs10844308. For the meta-analysis of all included studies, no genetic model indicated the significance of rs10844308 in PD risk under the fixed-effect model, and then, an analysis stratified by ethnicity was conducted. In the Chinese population, only the allele model (C vs. A) presented significantly increased PD risk with an OR of 1.18 (95% CI = 1.03–1.35). However, in the European population, all genetic models failed to show the significance of rs10844308 in PD risk (Table 3, Supplementary Figure).

**Table 3:** Meta-analysis of rs10844308 and rs143794289 in Parkinson's disease.

**rsID**	**Position**	**Minor allele**	**Major allele**	**Models**	**Chinese**				**European**				**Overall**				
					**OR**	***P*** **OR**	*I* ^2^	***p-*** **value**	**OR**	***P*** **OR**	*I* ^2^	***p-*** **value**	**OR**	***P*** **OR**	***P*** **FDR**	*I* ^2^	***p-*** **value**
rs10844308 (*N* = 6)	chr12:32854366	C	A	C vs. A	1.18 [1.03; 1.35]	**0.020**	0%	0.58	1.01 [0.98; 1.05]	0.398	0%	0.41	1.02 [0.99; 1.06]	0.168	0.280	33%	0.19
				CC + CA vs. AA	1.13 [0.97; 1.31]	0.111	9%	0.29	1.02 [0.99; 1.06]	0.224	27%	0.25	1.03 [0.99; 1.07]	0.114	0.285	26%	0.24
				CC vs. CA + AA	1.69 [0.88; 3.26]	0.116	0%	0.63	0.96 [0.86; 1.07]	0.445	0%	0.52	0.97 [0.87; 1.09]	0.640	0.800	6%	0.38
				CA vs. AA	1.10 [0.95; 1.28]	0.796	0%	0.33	1.03 [0.99; 1.07]	0.145	41%	0.16	1.03 [1.00; 1.07]	0.082	0.409	27%	0.23
				CC vs. AA	1.71 [0.89; 3.30]	0.108	0%	0.61	0.96 [0.86; 1.08]	0.526	0%	0.57	0.98 [0.88; 1.10]	0.740	0.740	2%	0.41
rs143794289 (*N* = 4)	chr12:32885540	A	G	A vs. G	0.76 [0.61; 0.94]	**0.012**	0%	0.56	0.80 [0.66; 0.97]	0.805	0%	0.63	0.80 [0.66; 0.97]	**0.023**	0.068	0%	0.65
				AA + AG vs. GG	0.76 [0.61; 0.95]	**0.015**	0%	0.63	0.95 [0.65; 1.39]	0.804	0%	0.62	0.81 [0.67; 0.98]	**0.027**	**0.027**	0%	0.68
				AG vs. GG	0.75 [0.60; 0.94]	**0.012**	0%	0.88	0.95 [0.65; 1.39]	0.804	0%	0.62	0.80 [0.66; 0.97]	**0.023**	**0.035**	0%	0.70

^*^N is the number of included studies in the meta-analysis; OR and POR values were calculated by Fisher's exact test.

The bold value means the *p* value < 0.05, suggesting significance.

The results of rs143794289 turned out to be more complicated but interesting. Unlike rs10844308, the homozygous mutation of rs143794289 was not found in public data, so we only applied the allele, dominant, and heterozygote genetic models to analyze the association between rs143794289 and susceptibility of PD. For rs143794289, we included four studies (the WGS cohort, the WES cohort, and two public cohorts) containing 15,072 patients and 20,541 controls. In the overall analysis, all genetic models showed no evidence of heterogeneity (*p* for heterogeneity > 0.1, *I*^2^ = 0) and presented significantly reduced risk of PD, with ORs of 0.80 (95% CI = 0.66–0.97), 0.81 (95% CI = 0.66–0.98), and 0.80 (95% CI = 0.66–0.97) for the allele, dominant, and heterozygote genetic models, respectively. After correction using the FDR method, the results of dominant and heterozygote genetic models remained significant (*p* = 0.027 and 0.035, respectively). Furthermore, in the Chinese population, all three genetic models show a protective role of rs143794289 in PD, while no significant result was found in the European population (Table 3, Supplementary Figure). All *p*-values from Egger's and Begg's tests were >0.05, indicating no publication bias in this meta-analysis.

## Discussion

*DNM1L* encodes DRP1, the main mitochondrial fission protein, which is strictly regulated to maintain the normal morphology of mitochondria (20). DRP1 is localized throughout the neuron, from dendrites and cell bodies to axons, maintaining the balance of mitochondrial fission and fusion in the neuron (21). In a previous study, researchers found that double knockout of *DNM1L* in mice is due to embryonic lethality, strongly suggesting that *DNM1L* is necessary for development and survival (22). In humans, *DNM1L* variants can cause lethal neonatal-onset encephalopathy or diverse degrees of cognitive impairment and epilepsy, indicating their essential role in the nervous system (23, 24). To date, no data have directly elucidated the relationship between *DNM1L* variants and PD, but numerous PD models showed dysregulation of DRP1. Therefore, it is meaningful and interesting to explore the genetic role of *DNM1L* in PD.

In this study, we comprehensively analyzed the rare and common variants in the *DNM1L* coding region in the Chinese mainland population. We identified 23 rare variants and 201 common variants totally in the *DNM1L* coding region. Almost all of these rare nonsynonymous variants were missense variants, five of which (p.V213A, p.L116Q, p.G120S, p.P255R, and p.P411L) were only found in patients with PD. Notably, we also found two splicing variants (c.987+2_987+3insA and c.988-1_988insTCT), which might cause the loss of function of DRP1. However, the rare variants we identified had yet to be previously reported. The protein of DNM1L has four domains, with most variants located in the middle and variable domains, while the splicing variant c.1636-1_1636insTCT is located in the GTPase effector domain (GED) (25). Since the impairment of DRP1 has been elucidated in numerous PD models, these variants are more functionally suggestive of being associated with PD risk and more experimental studies might be needed. We also conducted the burden analysis for the rare variants in two cohorts. Still, we found no significant relationship between *DNM1L* rare variants and PD, which revealed that the *DNM1L* gene might not play a crucial role in the risk of PD in the Chinese population.

With regard to common variants, we found that two common variants (rs10844308 and rs143794289) were significant in Fisher's exact test, with the former showing a risk effect and the latter showing a protective effect against PD. We noted inconsistent results for these two SNPs in the WES and WGS cohorts; therefore, we performed a meta-analysis combined with public data to further confirm their roles in PD risk. In a meta-analysis, the rs143794289 presented significantly reduced PD risk in the allele, dominant, and heterozygous genetic models, while rs10844308 showed no association with PD in all models. However, in the European population, the rs143794289 was not significantly associated with PD susceptibility. To note, rs143794289 is relatively rare in the European population, resulting in poor reliability in detecting a significant association. Thus, we cannot rule out a possible role for rs143794289 from the meta-analysis, and additional sample size is required for further validation.

To the best of our knowledge, this is the first study to explore the relationship between *DNM1L* variants and PD. However, our study has several limitations, including methodological bias, sample size, and sample population. First, different mutations of *DNM1L* might have diverse functions, but we did not verify these variants for functions, which might result in incorrect mutation grouping in the burden analysis. Some other forms of genetic changes such as copy number variation were not included in our study. Second, the sample size was still insufficient for rare variants analysis. Our cohorts contain only the Chinese mainland population, leading to discordance in sample ethnicities. Thus, enlarging sample size and adjusting the population substructure is possible to get a more reliable conclusion. We also found two novel splicing variants that may cause a loss of function but further functional validations in cell or animal models are needed. Moreover, almost *DNM1L*-related diseases are severe neurological disorders in children and teenagers, indicating that *DNM1L* is required for neurological development. Therefore, the pathogenic *DNM1L* variants are likely to cause widespread and severe damage to the nervous system rather than specific damage in dopaminergic neurons.

In conclusion, our study expanded the genetic spectrum of the *DNM1L* gene and preliminarily explored the *DNM1L* gene in PD. We identified several novel rare variants of the *DNM1L* gene and performed a meta-analysis for two potentially significant common variants. However, there was no evidence to support that the *DNM1L* gene's rare or common variants could increase the risk for PD in the mainland China's population. Therefore, larger sample sizes, more ethnicities, and more multivariate analysis methods will be needed.

## Data availability statement

The datasets presented in this study are deposited in https://db.cngb.org/cnsa/, accession number CNP0003925 (for WES cohort), and CNP0003902 (for WGS cohort).

## Ethics statement

The studies involving human participants were reviewed and approved by the Ethics Committee of Xiangya Hospital, Central South University (Approval No. 202005124). The patients/participants provided their written informed consent to participate in this study.

## Author contributions

JLiu: data curation, formal analysis, methodology, and writing—original draft. JH: methodology, writing—review and editing, and writing—original draft. YZ: data curation and methodology. HP: data curation and writing—review and editing. YW and ZL: data curation. QX, QS, JT, and XY: data curation and funding acquisition. JLi: funding acquisition and writing—review and editing. BT and JG: project administration, supervision, funding acquisition, and writing—review and editing. All authors contributed to the article and approved the submitted version.
