# Supplementary material for: Evaluating the association between DNM1L variants and Parkinson's disease in the Chinese population
Source: Front Neurol. 2023 Feb 24;14:1133449. doi: 10.3389/fneur.2023.1133449 (PMC9998701; doi:10.3389/fneur.2023.1133449)
Supplement: Supplementary file 1 [file Data_Sheet_1.docx]

**Supplementary Figure 1. Forest plot of ORs for the association between rs10844308 and PD risk under allele model ( C vs. A).**

**
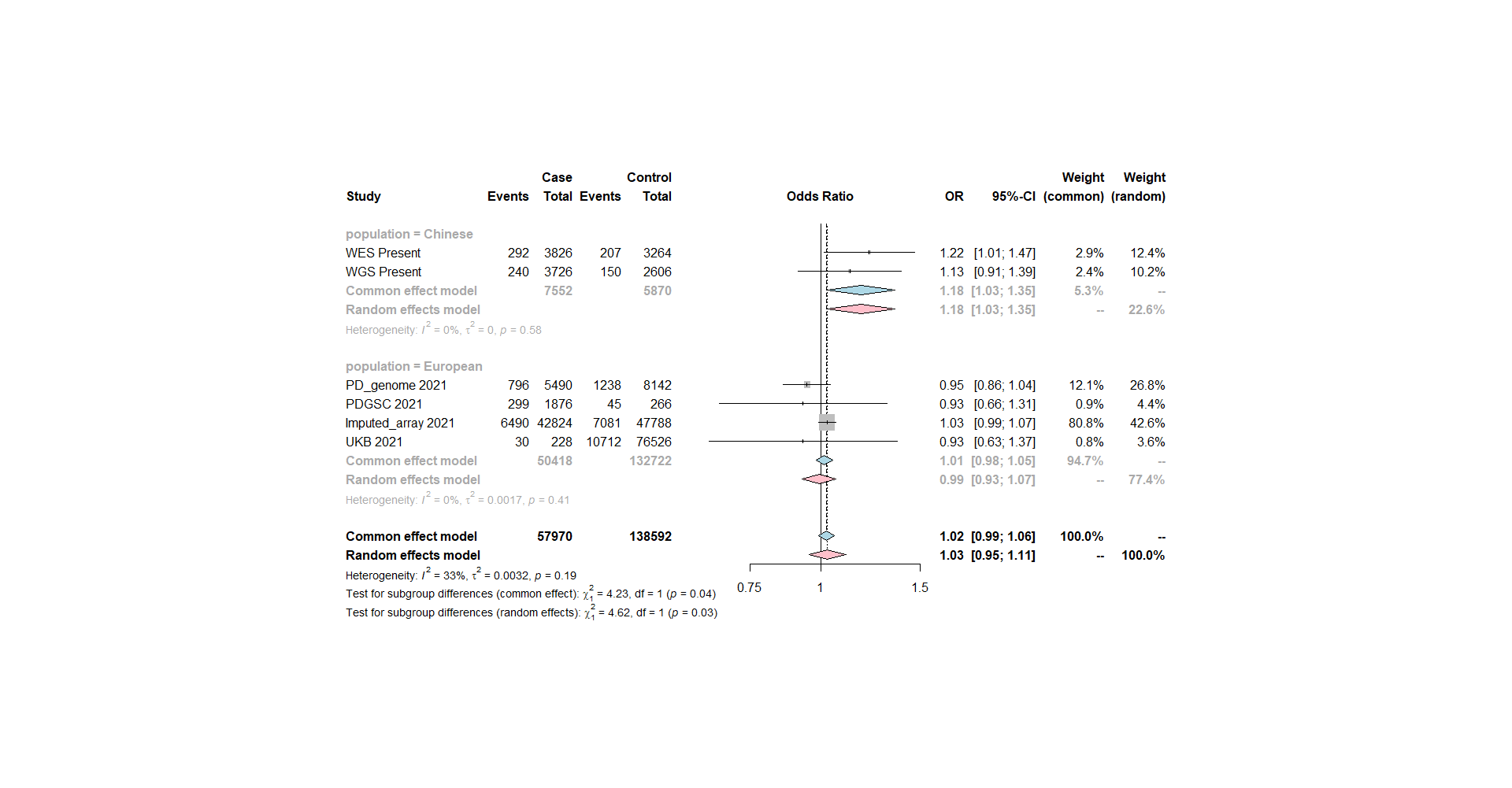
**

**Supplementary Figure 2. Forest plot of ORs for the association between rs10844308 and PD risk under dominant model ( CC + CA vs. AA).**

**
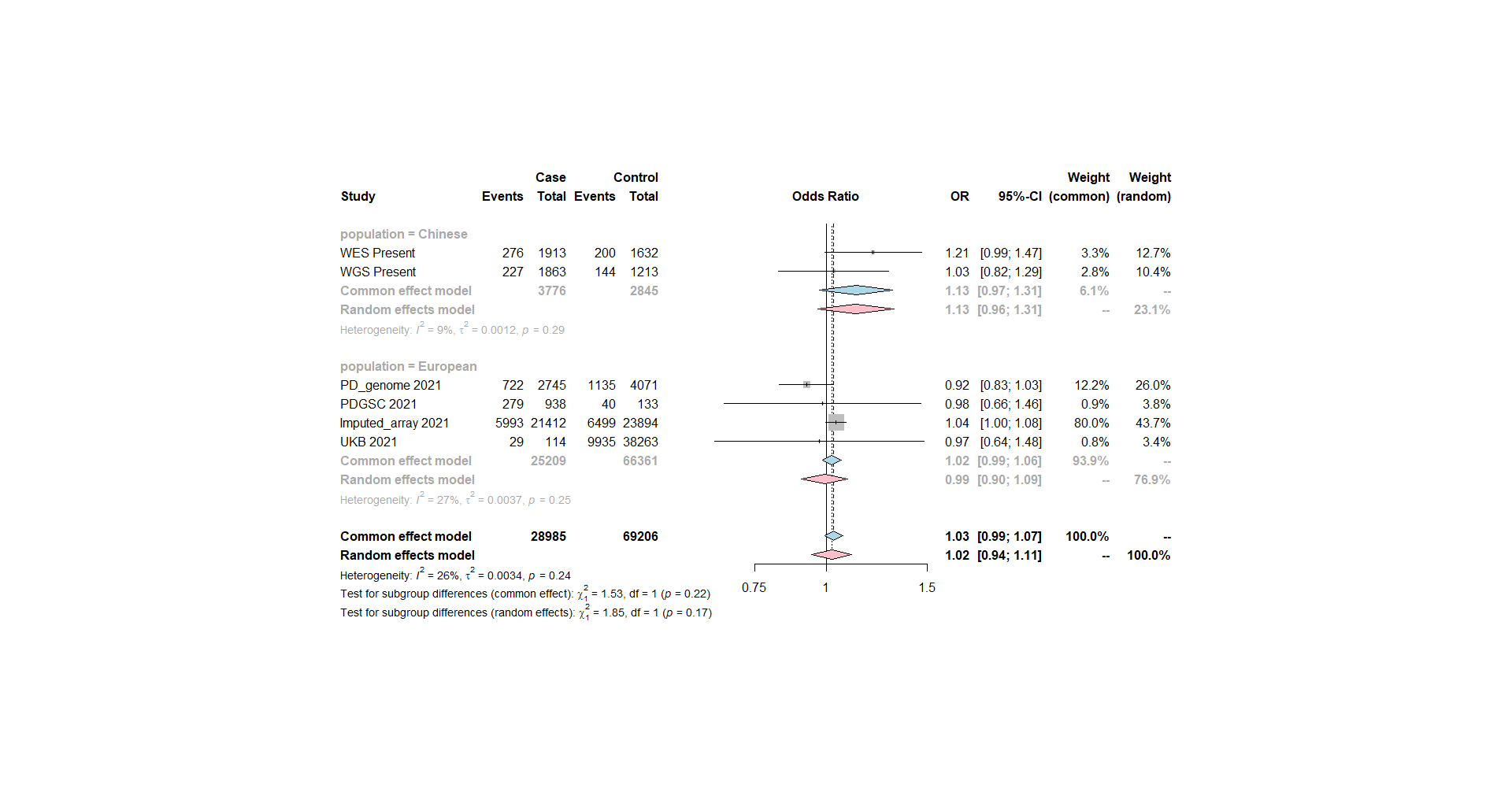
**

**Supplementary Figure 3. Forest plot of ORs for the association between rs10844308 and PD risk under recessive model ( CC vs. CA + AA).**

**
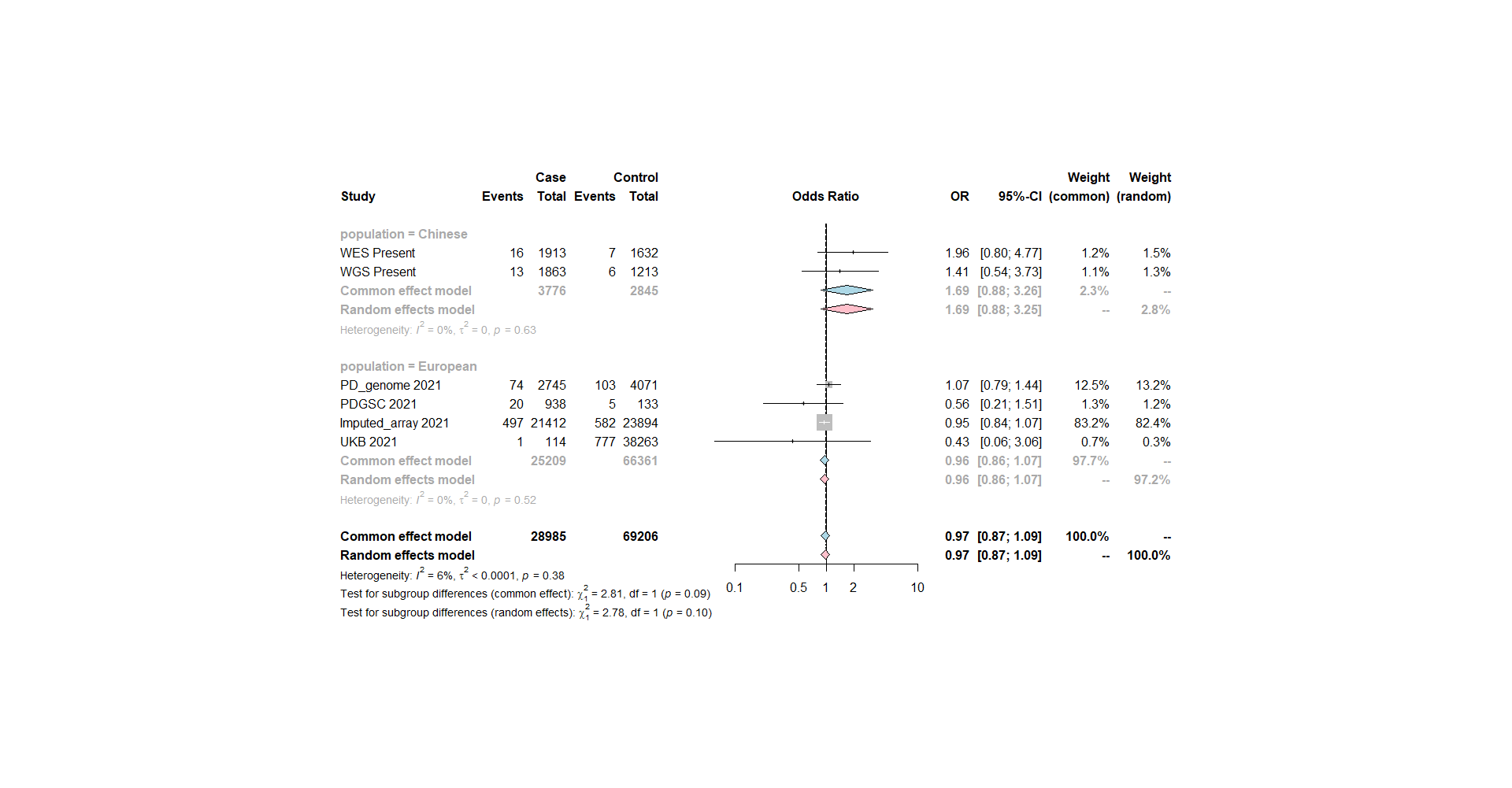
**

**Supplementary Figure 4. Forest plot of ORs for the association between rs10844308 and PD risk under heterozygote model ( CA vs. AA).**

**
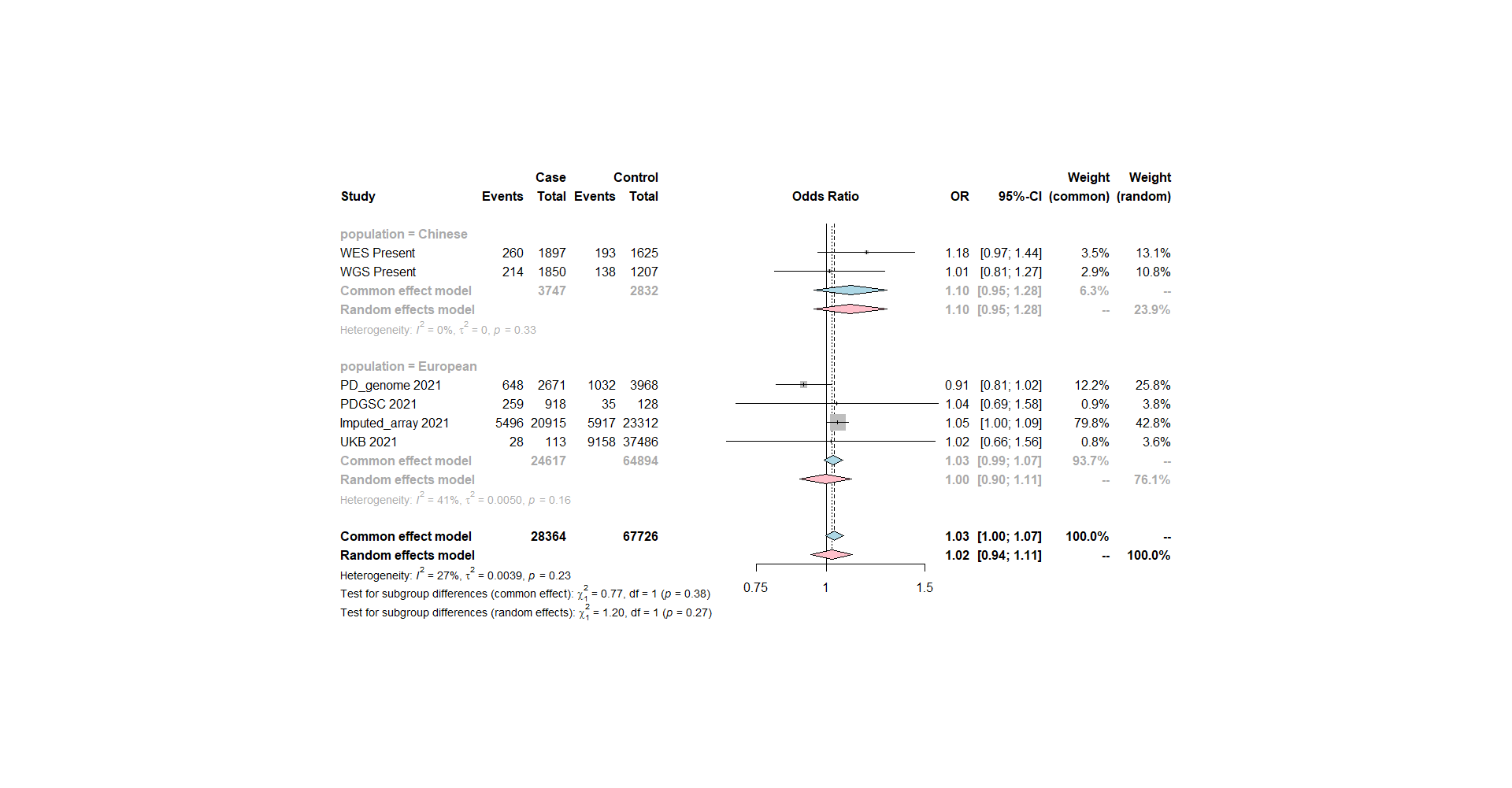
**

**Supplementary Figure 5. Forest plot of ORs for the association between rs10844308 and PD risk under homozygote model ( CC vs. AA).**

**
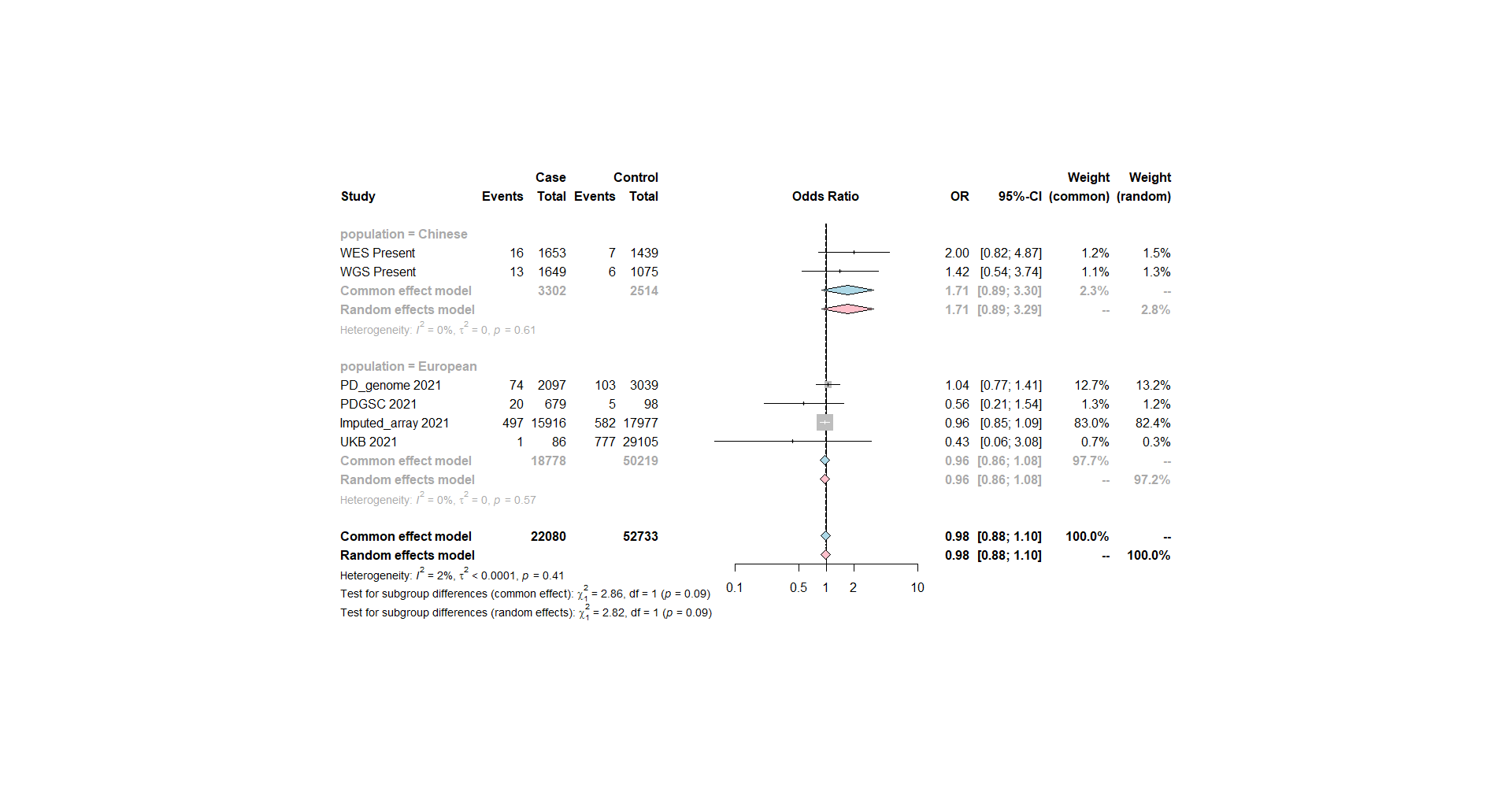
**

**Supplementary Figure 6. Forest plot of ORs for the association between rs143794289 and PD risk under allele model ( A vs. G).**

**
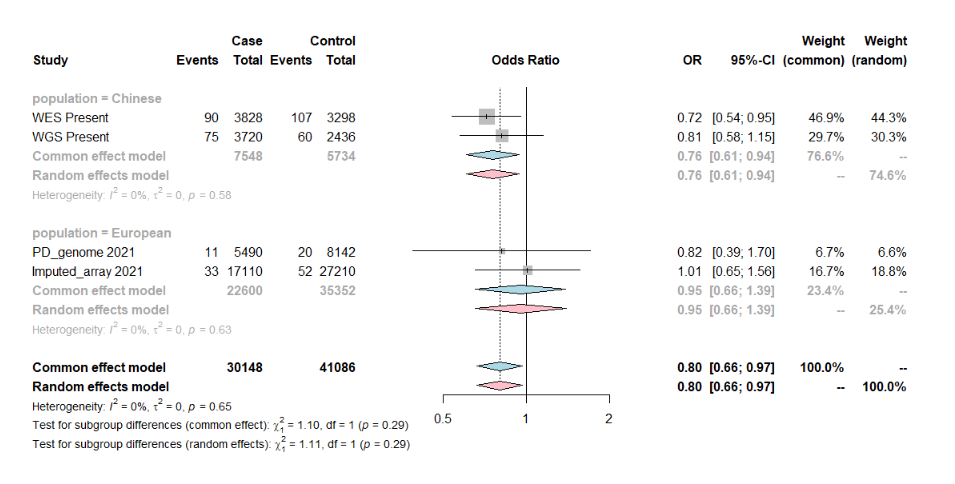
**

**Supplementary Figure 7. Forest plot of ORs for the association between rs143794289 and PD risk under dominant model ( AA + AG vs. GG)..**

**
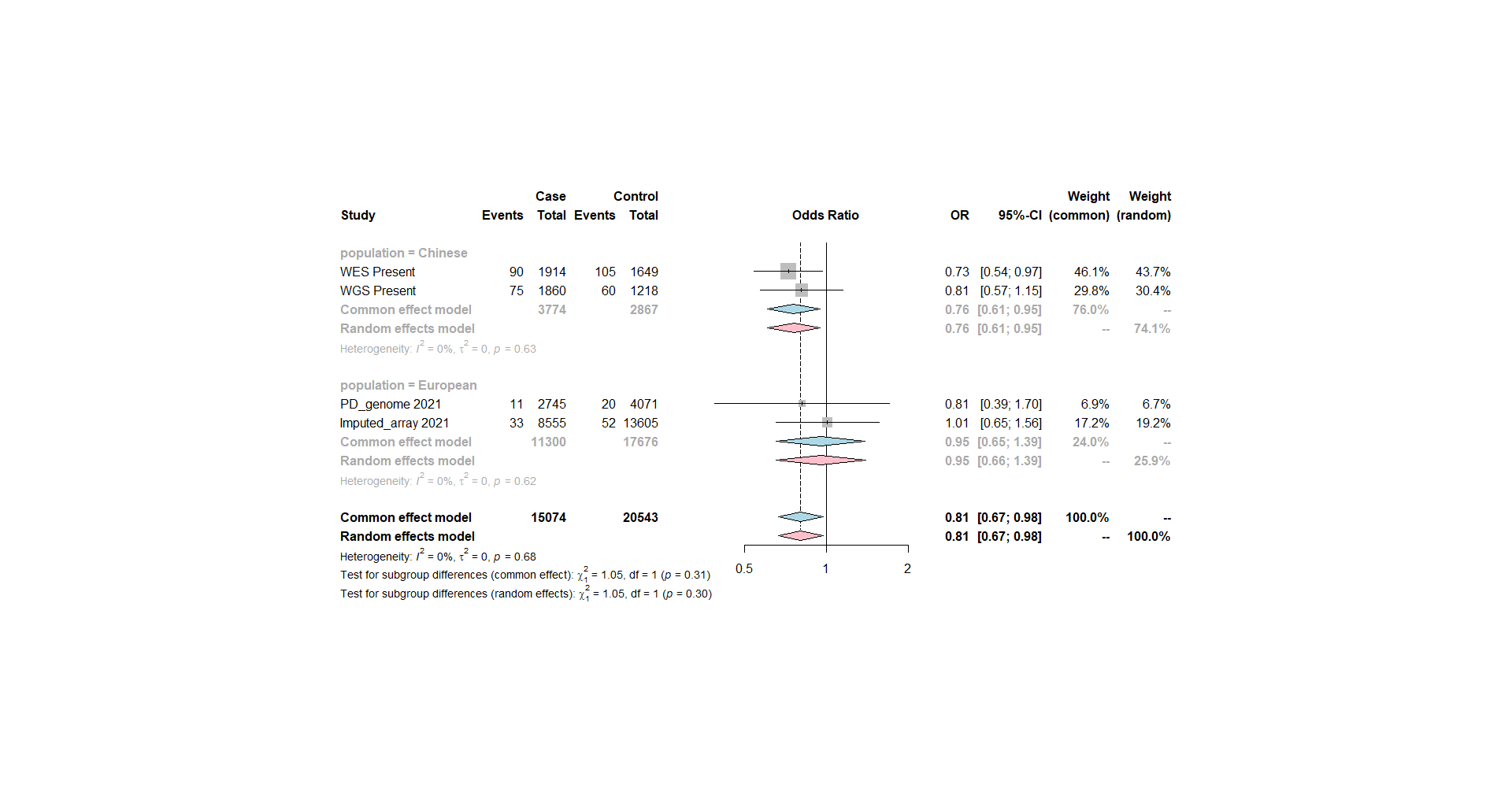
**

**Supplementary Figure 8. Forest plot of ORs for the association between rs143794289 and PD risk under heterozygote model ( AG vs. GG).**

**
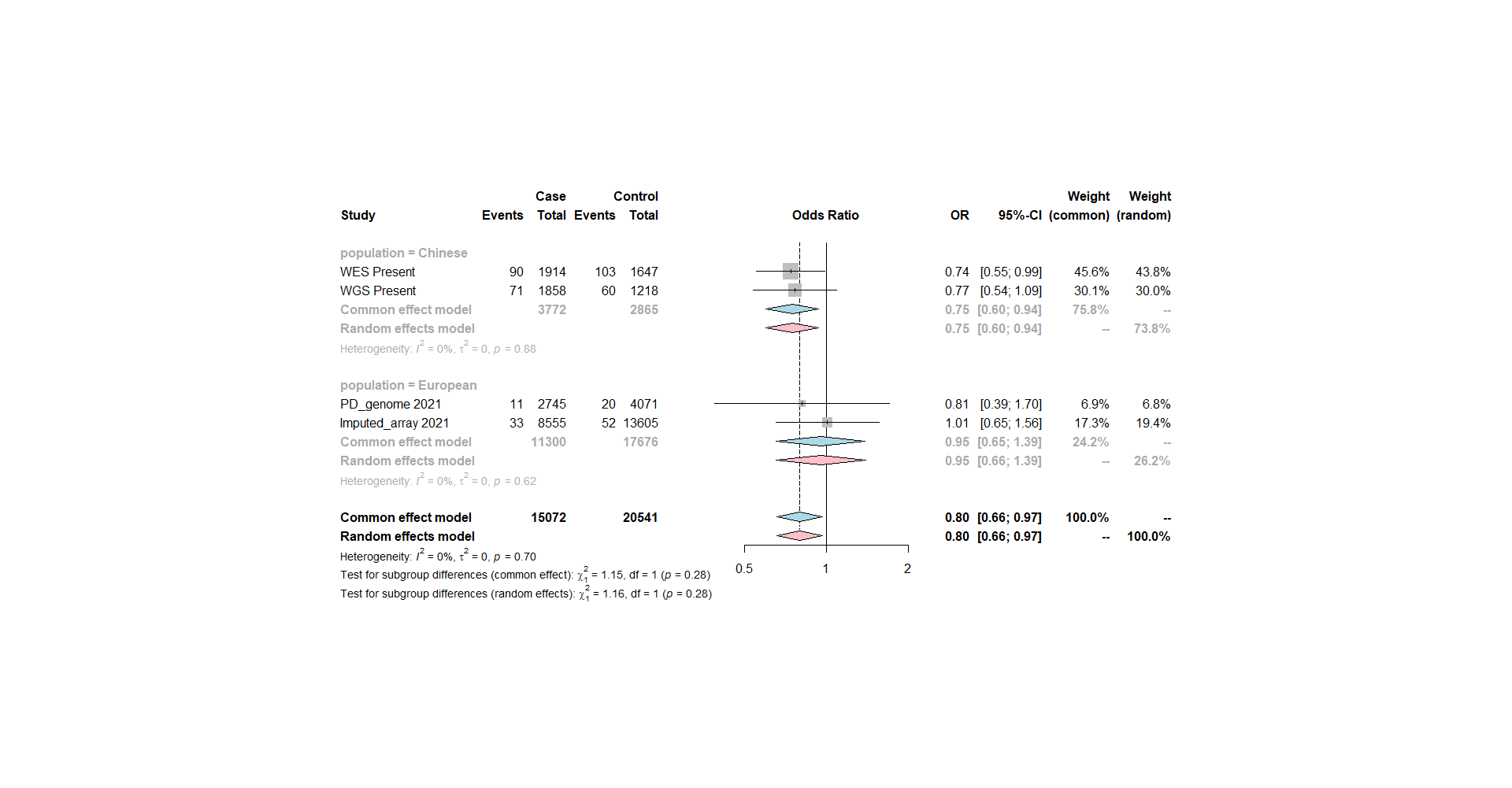
**
