# Supplementary material for: Evaluating the association between DNM1L variants and Parkinson's disease in the Chinese population
Source: Front Neurol. 2023 Feb 24;14:1133449. doi: 10.3389/fneur.2023.1133449 (PMC9998701; doi:10.3389/fneur.2023.1133449)
Supplement: Supplementary file 2 [file Table_1.docx]

Supplementary Table S1. Basic demographic characteristics of included subjects.

| **Cohorts** | **WES cohort** | | | |  | **WGS cohort** | |
| --- | --- | --- | --- | --- | --- | --- | --- |
|  | AD probands  (n = 327) | AR probands  (n = 150) | EOPD  (n = 1,440) | Control group 1  (n = 1,652) |  | LOPD  (n = 1,962) | Control group 2  (n = 1,279) |
| Age | 56.74±11.66 | 60.7±11.3 | 50.25±7.136 | 62.03±12.59 |  | 66.76±7.078 | 62.32±7.109 |
| Age at onset | 51.78±10.8 | 54.98±11.98 | 44.17±5.786 | - |  | 61.88±6.927 | - |
| Sex (male/female) | 180/147 | 82/68 | 786/654 | 795/857 |  | 984/978 | 613/666 |
| Genotyping method | WES | WES | WES | WES |  | WGS | WGS |

**Supplementary Table 2. Rare nonsynonymous variants of *TRADD* gene identified in our cohort**

| **Gene** | **Position (hg19)** | **Ref** | **Alt** | **AAChange** | **Consequence** | **gnomAD_exome_EAS ^a^** | **gnomAD_genome_EAS ^a^** | **ExAC_EAS ^a^** | **ReVe ^b^** | **WES cohort** | | **WGS cohort** | |
| --- | --- | --- | --- | --- | --- | --- | --- | --- | --- | --- | --- | --- | --- |
|  |  |  |  |  |  |  |  |  |  | **Case (n=1917)** | **Control (n=1652)** | **Case (n=1962)** | **Control (n=1279)** |
| *TRADD* | 16:67188568 | T | C | c.923A>G:p.N308S | missense | 0.0005 | - | 0.0004 | 0.134:T | 0 | 0 | 1 | 1 |
| *TRADD* | 16:67188728 | C | G | c.763G>C:p.E255Q | missense | 0 | - | 0 | 0.853:D | 1 | 0 | 0 | 0 |
| *TRADD* | 16:67188811 | C | A | c.680G>T:p.G227V | missense | - | - | - | 0.950:D | 1 | 0 | 0 | 0 |
| *TRADD* | 16:67188829 | G | T | c.662C>A:p.T221K | missense | 0.0033 | - | 0.0054 | 0.406:T | 0 | 0 | 1 | 0 |
| *TRADD* | 16:67189082 | G | A | c.545C>T:p.P182L | missense | - | - | - | 0.011:T | 0 | 0 | 0 | 1 |
| *TRADD* | 16:67189084 | C | A | c.543G>T:p.Q181H | missense | - | - | - | 0.185:T | 0 | 1 | 0 | 0 |
| *TRADD* | 16:67189097 | G | A | c.530C>T:p.S177L | missense | - | - | - | 0.110:T | 1 | 0 | 0 | 0 |
| *TRADD* | 16:67189122 | G | A | c.505C>T:p.R169W | missense | 0 | - | - | 0.349:T | 0 | 0 | 1 | 0 |
| *TRADD* | 16:67189137 | A | T | c.490T>A:p.C164S | missense | - | - | - | 0.424:T | 0 | 0 | 1 | 0 |
| *TRADD* | 16:67189167 | C | T | c.460G>A:p.E154K | missense | - | - | - | 0.536:T | 0 | 1 | 0 | 0 |
| *TRADD* | 16:67189315 | C | T | c.394G>A:p.E132K | missense | - | - | - | 0.564:T | 1 | 0 | 0 | 0 |
| *TRADD* | 16:67189320 | G | T | c.389C>A:p.A130E | missense | 0.0041 | 0.0025 | 0.0036 | 0.105:T | 12 | 13 | 9 | 11 |
| *TRADD* | 16:67189353 | C | A | c.356G>T:p.R119L | missense | - | - | - | 0.429:T | 0 | 0 | 1 | 0 |
| *TRADD* | 16:67189363 | G | T | c.346C>A:p.L116M | missense | 6.25E-05 | - | 0.0002 | 0.331:T | 1 | 0 | 1 | 0 |
| *TRADD* | 16:67189377 | G | A | c.332C>T:p.S111L | missense | 0 | 0 | 0 | 0.141:T | 0 | 1 | 0 | 0 |
| *TRADD* | 16:67189406 | C | G | c.303G>C:p.R101S | missense | - | - | - | 0.132:T | 1 | 0 | 0 | 0 |
| *TRADD* | 16:67189555 | T | C | c.154A>G:p.S52G | missense | 0.0035 | 0.0006 | 0.0049 | 0.317:T | 4 | 4 | 2 | 3 |
| *TRADD* | 16:67190466 | G | A | c.98C>T:p.A33V | missense | 0 | - | - | 0.115:T | 0 | 1 | 0 | 0 |
| *TRADD* | 16:67190494 | C | T | c.70G>A:p.D24N | missense | - | - | - | 0.130:T | 0 | 1 | 0 | 0 |

1. Variants minor allele frequencies from gnomAD_genome_EAS, gnomAD_exome_EAS and ExAC_EAS.
2. (The score of prediction software):(predictive results); D = Damaging; T = Tolerate.
