# Supplementary material for: Evaluating the association between DNM1L variants and Parkinson's disease in the Chinese population
Source: Front Neurol. 2023 Feb 24;14:1133449. doi: 10.3389/fneur.2023.1133449 (PMC9998701; doi:10.3389/fneur.2023.1133449)
Supplement: Supplementary file 3 [file Table_2.docx]

**Supplementary Table 2. Association analysis of common variants identified in the WES cohort.**

| **Position (hg19)** | **Minor allele** | **Major allele** | **Case (Hom/Het/Wild)** | **Control (Hom/Het/Wild)** | **MAF_AFF** | **MAF_**  **UNAFF** | **P - logistic** | **OR - logistic** | **P - fisher** | **OR - fisher** |
| --- | --- | --- | --- | --- | --- | --- | --- | --- | --- | --- |
| 12:32854366 | C | A | 16/260/1637 | 7/193/1432 | 0.076 | 0.063 | 0.789 | 1.040 | **0.036** | 1.220 |
| 12:32860302 | A | G | 16/261/1636 | 7/197/1425 | 0.077 | 0.065 | 0.940 | 1.011 | 0.057 | 1.198 |
| 12:32871481 | A | G | 16/207/1514 | 7/203/1435 | 0.069 | 0.066 | 0.429 | 0.889 | 0.663 | 1.046 |
| 12:32875406 | G | A | 16/246/1630 | 7/195/1427 | 0.073 | 0.064 | 0.908 | 1.017 | 0.132 | 1.157 |
| 12:32884741 | AAAAAAC | - | 10/180/1551 | 6/146/1341 | 0.057 | 0.053 | 0.373 | 0.857 | 0.445 | 1.091 |
| 12:32885540 | A | G | 0/90/1824 | 2/103/1544 | 0.024 | 0.032 | 0.200 | 0.747 | **0.025** | 0.718 |
| 12:32890161 | T | C | 16/252/1625 | 8/195/1415 | 0.075 | 0.065 | 0.859 | 0.975 | 0.112 | 1.163 |
| 12:32890685 | C | T | 1/40/1852 | 0/41/1601 | 0.011 | 0.012 | 0.440 | 0.765 | 0.658 | 0.887 |
| 12:32890912 | A | T | 34/411/1469 | 28/327/1293 | 0.125 | 0.116 | 0.912 | 0.988 | 0.259 | 1.088 |
| 12:32896365 | C | G | 16/267/1634 | 7/208/1436 | 0.078 | 0.067 | 0.974 | 0.995 | 0.083 | 1.173 |
| 12:32896366 | T | A | 16/267/1634 | 7/208/1436 | 0.078 | 0.067 | 0.974 | 0.995 | 0.083 | 1.173 |
| 12:32854366 | C | A | 16/260/1637 | 7/193/1432 | 0.076 | 0.063 | 0.789 | 1.040 | 0.036 | 1.220 |
| 12:32860302 | A | G | 16/261/1636 | 7/197/1425 | 0.077 | 0.065 | 0.940 | 1.011 | 0.057 | 1.198 |

The bold value means the *p* value < 0.05, suggesting significance.
