# Supplementary material for: Evaluating the association between DNM1L variants and Parkinson's disease in the Chinese population
Source: Front Neurol. 2023 Feb 24;14:1133449. doi: 10.3389/fneur.2023.1133449 (PMC9998701; doi:10.3389/fneur.2023.1133449)
Supplement: Supplementary file 4 [file Table_3.docx]

**Supplementary Table 3. Association analysis of common variants identified in the WGS cohort.**

| **Position (hg19)** | **Minor allele** | **Major allele** | **Case (Hom/Het/Wild)** | **Control (Hom/Het/Wild)** | **MAF_AFF** | **MAF_**  **UNAFF** | **P - logistic** | **OR - logistic** | **P - fisher** | **OR - fisher** |
| --- | --- | --- | --- | --- | --- | --- | --- | --- | --- | --- |
| 12:32830300 | C | G | 13/198/1623 | 5/143/1053 | 0.061 | 0.064 | 0.834 | 0.978 | 0.704 | 0.956 |
| 12:32830416 | A | C | 3/92/1682 | 0/56/1101 | 0.028 | 0.024 | 0.510 | 1.119 | 0.453 | 1.143 |
| 12:32830656 | T | C | 2/53/1751 | 1/37/1134 | 0.016 | 0.017 | 0.863 | 1.036 | 0.833 | 0.948 |
| 12:32831091 | C | T | 11/184/1612 | 6/136/1061 | 0.057 | 0.062 | 0.609 | 0.945 | 0.468 | 0.922 |
| 12:32831346 | T | G | 9/188/1623 | 5/136/1043 | 0.057 | 0.062 | 0.415 | 0.913 | 0.431 | 0.913 |
| 12:32832869 | C | G | 10/207/1670 | 6/143/1086 | 0.060 | 0.063 | 0.832 | 0.977 | 0.706 | 0.956 |
| 12:32832873 | T | C | 10/207/1674 | 6/144/1085 | 0.060 | 0.063 | 0.756 | 0.967 | 0.628 | 0.947 |
| 12:32832897 | G | T | 10/205/1678 | 6/143/1083 | 0.059 | 0.063 | 0.677 | 0.956 | 0.588 | 0.941 |
| 12:32832990 | A | T | 10/198/1641 | 5/129/1066 | 0.059 | 0.058 | 0.741 | 1.038 | 0.911 | 1.019 |
| 12:32833155 | T | G | 11/197/1623 | 6/136/1058 | 0.060 | 0.062 | 0.875 | 0.983 | 0.783 | 0.968 |
| 12:32834318 | C | T | 12/195/1588 | 6/132/1020 | 0.061 | 0.062 | 0.988 | 1.002 | 0.868 | 0.980 |
| 12:32834345 | A | C | 12/181/1589 | 6/126/1022 | 0.058 | 0.060 | 0.847 | 0.979 | 0.733 | 0.960 |
| 12:32834930 | A | C | 10/192/1580 | 3/133/1039 | 0.059 | 0.059 | 0.843 | 1.023 | 1.000 | 1.006 |
| 12:32835100 | T | C | 11/185/1569 | 6/128/1020 | 0.059 | 0.061 | 0.856 | 0.980 | 0.777 | 0.965 |
| 12:32835123 | A | G | 13/194/1588 | 8/122/1028 | 0.061 | 0.060 | 0.740 | 1.037 | 0.823 | 1.030 |
| 12:32835169 | C | T | 18/240/1570 | 9/162/1024 | 0.075 | 0.075 | 0.835 | 0.980 | 1.000 | 1.003 |
| 12:32835309 | G | A | 9/190/1631 | 7/134/1053 | 0.057 | 0.062 | 0.589 | 0.942 | 0.403 | 0.912 |
| 12:32835383 | A | C | 10/198/1647 | 7/126/1056 | 0.059 | 0.059 | 0.881 | 1.017 | 1.000 | 0.998 |
| 12:32835403 | T | C | 11/193/1638 | 8/127/1054 | 0.058 | 0.060 | 0.904 | 0.987 | 0.781 | 0.969 |
| 12:32835433 | G | T | 12/199/1648 | 7/132/1063 | 0.060 | 0.061 | 0.904 | 1.013 | 0.913 | 0.987 |
| 12:32835751 | G | A | 3/54/1823 | 0/39/1174 | 0.016 | 0.016 | 0.818 | 1.048 | 1.000 | 0.993 |
| 12:32836424 | T | G | 10/212/1583 | 5/138/1049 | 0.064 | 0.062 | 0.646 | 1.051 | 0.746 | 1.038 |
| 12:32836720 | T | A | 14/195/1600 | 5/140/1036 | 0.062 | 0.064 | 0.883 | 0.984 | 0.785 | 0.969 |
| 12:32837000 | A | C | 10/221/1653 | 6/146/1072 | 0.064 | 0.065 | 0.889 | 1.015 | 0.958 | 0.990 |
| 12:32837310 | C | T | 10/205/1677 | 6/135/1087 | 0.059 | 0.060 | 0.883 | 1.016 | 0.956 | 0.993 |
| 12:32837717 | T | C | 10/209/1661 | 7/140/1061 | 0.061 | 0.064 | 0.802 | 0.973 | 0.666 | 0.953 |
| 12:32837840 | T | C | 35/319/1440 | 14/220/923 | 0.108 | 0.107 | 0.704 | 1.033 | 0.898 | 1.013 |
| 12:32837978 | A | G | 11/193/1573 | 4/129/1012 | 0.061 | 0.060 | 0.768 | 1.034 | 0.955 | 1.012 |
| 12:32838579 | A | G | 10/217/1652 | 5/144/1068 | 0.063 | 0.063 | 0.951 | 1.007 | 1.000 | 0.997 |
| 12:32838761 | C | T | 10/200/1629 | 6/135/1059 | 0.060 | 0.061 | 0.971 | 0.996 | 0.826 | 0.975 |
| 12:32839272 | C | T | 10/206/1653 | 7/131/1069 | 0.060 | 0.060 | 0.808 | 1.027 | 1.000 | 1.007 |
| 12:32839293 | G | T | 11/199/1660 | 7/141/1067 | 0.059 | 0.064 | 0.557 | 0.939 | 0.479 | 0.922 |
| 12:32839316 | G | T | 10/205/1654 | 6/140/1075 | 0.060 | 0.062 | 0.840 | 0.978 | 0.745 | 0.965 |
| 12:32839537 | C | T | 10/203/1636 | 7/135/1066 | 0.060 | 0.062 | 0.954 | 0.994 | 0.827 | 0.976 |
| 12:32839755 | G | T | 10/204/1664 | 6/130/1065 | 0.060 | 0.059 | 0.753 | 1.035 | 0.956 | 1.009 |
| 12:32839788 | G | C | 11/191/1646 | 4/128/1067 | 0.058 | 0.057 | 0.705 | 1.044 | 0.910 | 1.017 |
| 12:32840047 | G | A | 2/37/1804 | 0/40/1164 | 0.011 | 0.017 | 0.071 | 0.670 | 0.085 | 0.666 |
| 12:32840180 | T | C | 11/198/1607 | 9/129/1040 | 0.061 | 0.062 | 0.958 | 0.994 | 0.783 | 0.969 |
| 12:32840244 | T | G | 10/195/1624 | 6/136/1049 | 0.059 | 0.062 | 0.783 | 0.970 | 0.618 | 0.943 |
| 12:32840284 | T | C | 11/192/1592 | 6/135/1034 | 0.060 | 0.063 | 0.842 | 0.978 | 0.657 | 0.950 |
| 12:32840436 | G | A | 10/195/1640 | 7/130/1058 | 0.058 | 0.060 | 0.947 | 0.993 | 0.781 | 0.965 |
| 12:32840528 | T | C | 12/195/1606 | 6/135/1039 | 0.060 | 0.062 | 0.966 | 0.995 | 0.783 | 0.968 |
| 12:32840788 | T | C | 11/204/1640 | 6/129/1061 | 0.061 | 0.059 | 0.758 | 1.035 | 0.783 | 1.036 |
| 12:32841060 | T | C | 11/192/1577 | 8/135/1039 | 0.060 | 0.064 | 0.641 | 0.951 | 0.581 | 0.937 |
| 12:32841106 | G | A | 10/206/1652 | 6/138/1065 | 0.060 | 0.062 | 0.916 | 0.989 | 0.828 | 0.974 |
| 12:32841110 | C | T | 10/212/1647 | 6/139/1057 | 0.062 | 0.063 | 0.960 | 1.005 | 0.914 | 0.987 |
| 12:32841160 | A | G | 11/222/1631 | 6/144/1076 | 0.065 | 0.064 | 0.675 | 1.045 | 0.792 | 1.031 |
| 12:32841294 | C | G | 11/200/1647 | 7/138/1066 | 0.060 | 0.063 | 0.753 | 0.967 | 0.624 | 0.949 |
| 12:32841407 | T | C | 2/70/1803 | 0/61/1157 | 0.020 | 0.025 | 0.131 | 0.766 | 0.182 | 0.784 |
| 12:32841526 | A | G | 11/183/1637 | 8/141/1066 | 0.056 | 0.065 | 0.209 | 0.873 | 0.167 | 0.859 |
| 12:32841566 | G | C | 3/107/1770 | 1/63/1163 | 0.030 | 0.026 | 0.433 | 1.133 | 0.437 | 1.139 |
| 12:32842217 | C | T | 9/222/1664 | 6/135/1072 | 0.063 | 0.061 | 0.564 | 1.065 | 0.707 | 1.048 |
| 12:32842242 | T | C | 8/219/1637 | 6/134/1062 | 0.063 | 0.061 | 0.623 | 1.055 | 0.745 | 1.040 |
| 12:32842262 | C | G | 9/210/1637 | 7/135/1059 | 0.061 | 0.062 | 0.912 | 1.012 | 0.957 | 0.990 |
| 12:32842323 | A | G | 11/188/1599 | 6/127/1048 | 0.058 | 0.059 | 0.935 | 1.009 | 0.955 | 0.992 |
| 12:32842876 | C | G | 39/330/1479 | 13/230/949 | 0.110 | 0.107 | 0.561 | 1.050 | 0.736 | 1.031 |
| 12:32842969 | G | A | 10/192/1655 | 5/134/1063 | 0.057 | 0.060 | 0.787 | 0.970 | 0.655 | 0.950 |
| 12:32843021 | C | T | 11/185/1643 | 7/134/1055 | 0.056 | 0.062 | 0.525 | 0.933 | 0.371 | 0.904 |
| 12:32843640 | T | C | 10/212/1622 | 5/138/1072 | 0.063 | 0.061 | 0.687 | 1.045 | 0.787 | 1.035 |
| 12:32843771 | A | G | 9/197/1633 | 7/147/1058 | 0.058 | 0.066 | 0.250 | 0.884 | 0.211 | 0.873 |
| 12:32843868 | G | C | 8/204/1634 | 7/132/1061 | 0.060 | 0.061 | 0.954 | 0.994 | 0.869 | 0.978 |
| 12:32844086 | T | C | 9/208/1636 | 7/131/1057 | 0.061 | 0.061 | 0.842 | 1.022 | 1.000 | 1.005 |
| 12:32844176 | A | G | 11/207/1616 | 8/131/1051 | 0.062 | 0.062 | 0.778 | 1.031 | 0.957 | 1.012 |
| 12:32844213 | A | G | 10/203/1629 | 7/134/1048 | 0.061 | 0.062 | 0.998 | 1.000 | 0.784 | 0.971 |
| 12:32844234 | A | G | 11/196/1626 | 6/141/1059 | 0.059 | 0.063 | 0.668 | 0.955 | 0.547 | 0.934 |
| 12:32844670 | A | T | 11/208/1612 | 6/126/1038 | 0.063 | 0.059 | 0.468 | 1.083 | 0.581 | 1.069 |
| 12:32844706 | G | A | 11/208/1629 | 8/130/1047 | 0.062 | 0.062 | 0.802 | 1.027 | 0.957 | 1.011 |
| 12:32844767 | A | G | 13/202/1644 | 7/135/1041 | 0.061 | 0.063 | 0.951 | 0.993 | 0.827 | 0.972 |
| 12:32844970 | C | T | 9/211/1623 | 5/134/1043 | 0.062 | 0.061 | 0.748 | 1.036 | 0.870 | 1.021 |
| 12:32845482 | G | C | 10/198/1632 | 7/140/1055 | 0.059 | 0.064 | 0.565 | 0.940 | 0.444 | 0.920 |
| 12:32845606 | G | A | 10/204/1637 | 6/134/1062 | 0.061 | 0.061 | 0.883 | 1.016 | 1.000 | 0.996 |
| 12:32845999 | C | T | 10/210/1658 | 6/142/1070 | 0.061 | 0.063 | 0.820 | 0.976 | 0.747 | 0.967 |
| 12:32846272 | A | G | 10/196/1606 | 6/134/1063 | 0.060 | 0.061 | 0.982 | 0.998 | 0.868 | 0.981 |
| 12:32846476 | T | A | 10/193/1662 | 5/137/1088 | 0.057 | 0.060 | 0.868 | 0.982 | 0.658 | 0.953 |
| 12:32846696 | T | C | 10/189/1628 | 7/129/1042 | 0.057 | 0.061 | 0.690 | 0.957 | 0.574 | 0.939 |
| 12:32846930 | C | T | 3/100/1784 | 0/51/1178 | 0.028 | 0.021 | 0.081 | 1.352 | 0.082 | 1.364 |
| 12:32846983 | G | A | 10/204/1646 | 6/129/1058 | 0.060 | 0.059 | 0.747 | 1.036 | 0.868 | 1.020 |
| 12:32847164 | C | T | 8/204/1614 | 6/139/1065 | 0.060 | 0.062 | 0.809 | 0.974 | 0.743 | 0.963 |
| 12:32847348 | G | A | 10/226/1657 | 6/142/1080 | 0.065 | 0.063 | 0.620 | 1.054 | 0.751 | 1.039 |
| 12:32847412 | C | T | 10/216/1667 | 7/148/1074 | 0.062 | 0.066 | 0.730 | 0.964 | 0.596 | 0.942 |
| 12:32847501 | T | C | 8/193/1654 | 6/132/1060 | 0.056 | 0.060 | 0.667 | 0.953 | 0.537 | 0.934 |
| 12:32847609 | A | T | 9/192/1650 | 5/132/1061 | 0.057 | 0.059 | 0.800 | 0.972 | 0.694 | 0.955 |
| 12:32847704 | A | C | 9/203/1645 | 7/130/1056 | 0.060 | 0.060 | 0.998 | 1.000 | 0.912 | 0.985 |
| 12:32847747 | A | T | 9/200/1643 | 7/137/1058 | 0.059 | 0.063 | 0.621 | 0.948 | 0.546 | 0.933 |
| 12:32847862 | T | C | 9/207/1604 | 7/128/1048 | 0.062 | 0.060 | 0.633 | 1.054 | 0.826 | 1.032 |
| 12:32848072 | T | C | 11/200/1632 | 6/137/1047 | 0.060 | 0.063 | 0.849 | 0.979 | 0.701 | 0.960 |
| 12:32848353 | T | C | 9/205/1640 | 6/139/1060 | 0.060 | 0.063 | 0.859 | 0.981 | 0.702 | 0.957 |
| 12:32848525 | T | G | 10/209/1598 | 7/138/1033 | 0.063 | 0.065 | 0.940 | 0.992 | 0.829 | 0.975 |
| 12:32848579 | T | C | 11/193/1602 | 6/137/1034 | 0.060 | 0.063 | 0.693 | 0.958 | 0.580 | 0.937 |
| 12:32849237 | G | A | 9/204/1553 | 7/127/1018 | 0.063 | 0.061 | 0.671 | 1.048 | 0.825 | 1.029 |
| 12:32850212 | T | A | 12/197/1618 | 5/130/1039 | 0.060 | 0.060 | 0.812 | 1.027 | 0.912 | 1.015 |
| 12:32850423 | G | C | 10/206/1626 | 6/140/1062 | 0.061 | 0.063 | 0.891 | 0.985 | 0.828 | 0.974 |
| 12:32851313 | T | G | 0/49/1824 | 1/43/1170 | 0.013 | 0.019 | 0.094 | 0.704 | 0.090 | 0.702 |
| 12:32851380 | C | T | 11/206/1635 | 7/131/1056 | 0.062 | 0.061 | 0.821 | 1.025 | 0.913 | 1.015 |
| 12:32851417 | C | T | 11/198/1630 | 6/141/1054 | 0.060 | 0.064 | 0.640 | 0.951 | 0.548 | 0.935 |
| 12:32851588 | A | G | 10/208/1638 | 6/130/1059 | 0.061 | 0.059 | 0.597 | 1.060 | 0.784 | 1.036 |
| 12:32851833 | T | C | 12/213/1618 | 6/130/1062 | 0.064 | 0.059 | 0.277 | 1.126 | 0.448 | 1.091 |
| 12:32852009 | T | C | 10/210/1630 | 7/138/1044 | 0.062 | 0.064 | 0.841 | 0.979 | 0.787 | 0.971 |
| 12:32852205 | T | C | 3/100/1719 | 1/62/1115 | 0.029 | 0.027 | 0.705 | 1.063 | 0.691 | 1.073 |
| 12:32852206 | A | G | 10/196/1611 | 6/127/1061 | 0.059 | 0.058 | 0.796 | 1.029 | 0.867 | 1.022 |
| 12:32852279 | G | T | 12/211/1637 | 7/135/1054 | 0.063 | 0.062 | 0.803 | 1.027 | 0.914 | 1.015 |
| 12:32852880 | G | A | 10/203/1625 | 6/142/1055 | 0.061 | 0.064 | 0.694 | 0.958 | 0.625 | 0.944 |
| 12:32852949 | G | A | 11/206/1649 | 6/136/1072 | 0.061 | 0.061 | 0.906 | 1.013 | 1.000 | 1.002 |
| 12:32853141 | G | C | 13/211/1599 | 7/140/1038 | 0.065 | 0.065 | 0.919 | 1.011 | 1.000 | 1.000 |
| 12:32853157 | G | A | 38/324/1424 | 13/205/923 | 0.112 | 0.101 | 0.143 | 1.134 | 0.210 | 1.120 |
| 12:32853711 | G | A | 11/190/1603 | 6/133/1035 | 0.059 | 0.062 | 0.780 | 0.970 | 0.655 | 0.949 |
| 12:32853755 | G | A | 11/197/1655 | 7/131/1067 | 0.059 | 0.060 | 0.949 | 0.993 | 0.825 | 0.976 |
| 12:32854082 | T | C | 13/205/1613 | 6/136/1039 | 0.063 | 0.063 | 0.862 | 1.019 | 0.957 | 1.007 |
| 12:32854366 | C | A | 13/214/1636 | 6/138/1069 | 0.064 | 0.062 | 0.624 | 1.054 | 0.708 | 1.045 |
| 12:32855337 | A | G | 10/200/1602 | 7/129/1036 | 0.061 | 0.061 | 0.959 | 1.006 | 0.956 | 0.995 |
| 12:32855372 | G | A | 1/53/1813 | 0/36/1170 | 0.015 | 0.015 | 0.717 | 1.083 | 1.000 | 0.987 |
| 12:32855435 | G | A | 11/187/1645 | 5/136/1072 | 0.057 | 0.060 | 0.716 | 0.961 | 0.577 | 0.939 |
| 12:32856311 | T | G | 11/190/1604 | 6/138/1055 | 0.059 | 0.063 | 0.674 | 0.955 | 0.543 | 0.935 |
| 12:32856415 | T | C | 10/204/1611 | 7/133/1050 | 0.061 | 0.062 | 0.929 | 1.010 | 0.956 | 0.993 |
| 12:32857300 | A | G | 18/243/1546 | 8/171/997 | 0.077 | 0.080 | 1.000 | 1.000 | 0.767 | 0.969 |
| 12:32857787 | G | A | 2/82/1815 | 0/54/1181 | 0.023 | 0.022 | 0.824 | 1.040 | 0.862 | 1.037 |
| 12:32858414 | G | A | 4/104/1785 | 1/68/1167 | 0.030 | 0.028 | 0.784 | 1.043 | 0.818 | 1.046 |
| 12:32859757 | T | C | 11/198/1636 | 6/144/1055 | 0.060 | 0.065 | 0.557 | 0.939 | 0.415 | 0.916 |
| 12:32860302 | A | G | 9/217/1628 | 6/136/1050 | 0.063 | 0.062 | 0.735 | 1.037 | 0.871 | 1.022 |
| 12:32860746 | T | G | 11/201/1618 | 5/137/1044 | 0.061 | 0.062 | 0.963 | 0.995 | 0.869 | 0.982 |
| 12:32861409 | C | T | 4/94/1752 | 2/72/1150 | 0.028 | 0.031 | 0.423 | 0.885 | 0.438 | 0.885 |
| 12:32861611 | A | T | 9/170/1644 | 2/104/1096 | 0.052 | 0.045 | 0.252 | 1.153 | 0.248 | 1.156 |
| 12:32862309 | T | G | 9/203/1651 | 5/138/1074 | 0.059 | 0.061 | 0.940 | 0.992 | 0.826 | 0.974 |
| 12:32863521 | T | C | 12/185/1592 | 5/127/1026 | 0.058 | 0.059 | 0.982 | 0.998 | 0.910 | 0.987 |
| 12:32863625 | G | A | 10/188/1605 | 5/135/1029 | 0.058 | 0.062 | 0.596 | 0.943 | 0.501 | 0.926 |
| 12:32863824 | C | T | 12/199/1563 | 7/131/1023 | 0.063 | 0.062 | 0.813 | 1.026 | 1.000 | 1.007 |
| 12:32864459 | T | C | 11/207/1635 | 8/122/1060 | 0.062 | 0.058 | 0.442 | 1.088 | 0.581 | 1.070 |
| 12:32864527 | A | G | 12/211/1643 | 6/131/1049 | 0.063 | 0.060 | 0.478 | 1.080 | 0.703 | 1.047 |
| 12:32866399 | A | G | 9/205/1639 | 7/135/1061 | 0.060 | 0.062 | 0.902 | 0.987 | 0.784 | 0.970 |
| 12:32866403 | C | T | 9/201/1658 | 7/134/1073 | 0.059 | 0.061 | 0.834 | 0.977 | 0.700 | 0.959 |
| 12:32866516 | T | A | 11/201/1658 | 7/141/1058 | 0.060 | 0.064 | 0.530 | 0.935 | 0.480 | 0.923 |
| 12:32866557 | A | G | 11/200/1584 | 7/141/1012 | 0.062 | 0.067 | 0.520 | 0.933 | 0.446 | 0.921 |
| 12:32866658 | G | C | 11/209/1625 | 8/144/1056 | 0.063 | 0.066 | 0.691 | 0.959 | 0.593 | 0.942 |
| 12:32866812 | T | C | 11/208/1632 | 7/130/1048 | 0.062 | 0.061 | 0.737 | 1.037 | 0.870 | 1.024 |
| 12:32867035 | A | C | 0/63/1751 | 1/39/1141 | 0.017 | 0.017 | 0.868 | 0.967 | 1.000 | 1.000 |
| 12:32867467 | A | G | 11/180/1614 | 8/120/1034 | 0.056 | 0.059 | 0.710 | 0.959 | 0.688 | 0.954 |
| 12:32867559 | T | A | 9/206/1671 | 6/140/1072 | 0.059 | 0.062 | 0.710 | 0.961 | 0.624 | 0.949 |
| 12:32868238 | G | A | 10/216/1645 | 7/141/1065 | 0.063 | 0.064 | 0.998 | 1.000 | 0.915 | 0.986 |
| 12:32868267 | C | T | 8/111/1771 | 1/102/1125 | 0.034 | 0.042 | 0.122 | 0.813 | 0.075 | 0.786 |
| 12:32869305 | A | G | 11/203/1638 | 6/139/1072 | 0.061 | 0.062 | 0.961 | 0.995 | 0.870 | 0.978 |
| 12:32871326 | C | T | 36/349/1437 | 25/222/939 | 0.116 | 0.115 | 0.844 | 1.016 | 0.934 | 1.008 |
| 12:32871481 | A | G | 10/211/1656 | 6/138/1068 | 0.062 | 0.062 | 0.928 | 1.010 | 0.957 | 0.994 |
| 12:32871860 | A | G | 10/194/1613 | 5/126/1040 | 0.059 | 0.058 | 0.748 | 1.037 | 0.910 | 1.015 |
| 12:32872792 | A | T | 10/193/1621 | 6/137/1042 | 0.058 | 0.063 | 0.616 | 0.946 | 0.471 | 0.924 |
| 12:32873856 | T | G | 1/189/1652 | 4/114/1081 | 0.052 | 0.051 | 0.988 | 1.002 | 0.906 | 1.020 |
| 12:32873857 | A | T | 1/189/1660 | 4/114/1084 | 0.052 | 0.051 | 0.988 | 0.998 | 0.906 | 1.018 |
| 12:32873916 | T | C | 11/211/1616 | 8/133/1029 | 0.063 | 0.064 | 0.906 | 1.013 | 0.957 | 0.995 |
| 12:32874324 | T | G | 11/191/1642 | 6/132/1052 | 0.058 | 0.061 | 0.750 | 0.966 | 0.655 | 0.952 |
| 12:32874982 | T | C | 1/75/1791 | 3/44/1175 | 0.021 | 0.020 | 0.944 | 0.987 | 1.000 | 1.008 |
| 12:32875052 | C | T | 11/190/1598 | 6/138/1034 | 0.059 | 0.064 | 0.556 | 0.938 | 0.471 | 0.921 |
| 12:32875406 | G | A | 10/202/1645 | 6/130/1064 | 0.060 | 0.059 | 0.796 | 1.029 | 0.956 | 1.011 |
| 12:32876331 | G | C | 11/198/1615 | 7/136/1053 | 0.060 | 0.063 | 0.832 | 0.977 | 0.701 | 0.959 |
| 12:32877072 | T | C | 10/201/1642 | 6/136/1060 | 0.060 | 0.062 | 0.908 | 0.988 | 0.784 | 0.967 |
| 12:32877842 | G | A | 11/193/1583 | 6/133/1043 | 0.060 | 0.061 | 0.905 | 0.987 | 0.868 | 0.980 |
| 12:32878475 | G | A | 12/198/1657 | 7/136/1069 | 0.059 | 0.062 | 0.740 | 0.965 | 0.702 | 0.958 |
| 12:32878563 | C | T | 37/328/1465 | 16/229/950 | 0.110 | 0.109 | 0.736 | 1.028 | 0.966 | 1.006 |
| 12:32878800 | C | A | 11/195/1628 | 6/144/1058 | 0.059 | 0.065 | 0.449 | 0.922 | 0.413 | 0.911 |
| 12:32878869 | A | G | 13/187/1573 | 7/124/1016 | 0.060 | 0.060 | 0.931 | 1.010 | 1.000 | 0.998 |
| 12:32878934 | G | A | 10/190/1627 | 7/127/1050 | 0.057 | 0.060 | 0.887 | 0.984 | 0.736 | 0.963 |
| 12:32879346 | A | C | 10/195/1607 | 8/129/1030 | 0.059 | 0.062 | 0.795 | 0.972 | 0.656 | 0.952 |
| 12:32879543 | G | T | 1/55/1800 | 0/39/1176 | 0.015 | 0.016 | 0.889 | 1.030 | 0.834 | 0.956 |
| 12:32879976 | A | G | 13/198/1607 | 6/117/1049 | 0.062 | 0.055 | 0.235 | 1.143 | 0.312 | 1.127 |
| 12:32881458 | T | A | 10/200/1634 | 6/123/1055 | 0.060 | 0.057 | 0.530 | 1.073 | 0.695 | 1.049 |
| 12:32881594 | G | A | 57/411/1317 | 30/291/836 | 0.147 | 0.152 | 0.792 | 0.981 | 0.626 | 0.964 |
| 12:32881842 | T | C | 10/199/1627 | 6/131/1056 | 0.060 | 0.060 | 0.904 | 1.013 | 0.956 | 0.995 |
| 12:32881960 | T | C | 10/206/1628 | 6/136/1055 | 0.061 | 0.062 | 0.979 | 1.003 | 0.956 | 0.991 |
| 12:32882003 | A | G | 8/204/1659 | 6/136/1074 | 0.059 | 0.061 | 0.886 | 0.984 | 0.742 | 0.964 |
| 12:32882160 | T | C | 0/59/1781 | 1/41/1165 | 0.016 | 0.018 | 0.768 | 0.941 | 0.611 | 0.898 |
| 12:32882562 | T | G | 9/200/1559 | 6/139/1028 | 0.062 | 0.064 | 0.761 | 0.967 | 0.701 | 0.955 |
| 12:32885110 | A | G | 12/202/1625 | 6/145/1038 | 0.061 | 0.066 | 0.657 | 0.954 | 0.483 | 0.926 |
| 12:32885540 | A | G | 2/71/1787 | 0/60/1158 | 0.020 | 0.025 | 0.194 | 0.795 | 0.248 | 0.815 |
| 12:32886015 | C | T | 11/216/1636 | 6/145/1063 | 0.064 | 0.065 | 0.941 | 1.008 | 0.915 | 0.987 |
| 12:32886193 | T | C | 12/194/1640 | 5/143/1065 | 0.059 | 0.063 | 0.582 | 0.942 | 0.547 | 0.932 |
| 12:32887210 | G | A | 10/203/1640 | 6/137/1066 | 0.060 | 0.062 | 0.997 | 1.000 | 0.827 | 0.975 |
| 12:32887272 | C | T | 10/215/1643 | 5/140/1060 | 0.063 | 0.062 | 0.761 | 1.033 | 0.957 | 1.011 |
| 12:32889902 | C | T | 4/110/1776 | 1/59/1150 | 0.031 | 0.025 | 0.223 | 1.215 | 0.186 | 1.246 |
| 12:32889911 | T | C | 33/323/1481 | 14/214/960 | 0.106 | 0.102 | 0.692 | 1.034 | 0.636 | 1.044 |
| 12:32890161 | T | C | 10/196/1630 | 6/133/1060 | 0.059 | 0.060 | 0.889 | 0.985 | 0.824 | 0.971 |
| 12:328 90343 | A | G | 10/193/1625 | 6/131/1057 | 0.058 | 0.060 | 0.885 | 0.984 | 0.823 | 0.971 |
| 12:32890551 | C | T | 3/95/1763 | 1/61/1165 | 0.027 | 0.026 | 0.747 | 1.054 | 0.747 | 1.059 |
| 12:32890912 | A | T | 34/313/1487 | 13/234/944 | 0.104 | 0.109 | 0.720 | 0.970 | 0.522 | 0.946 |
| 12:32892678 | T | C | 14/202/1633 | 6/137/1048 | 0.062 | 0.063 | 0.878 | 1.017 | 0.957 | 0.994 |
| 12:32892823 | C | G | 5/135/1716 | 2/76/1125 | 0.039 | 0.033 | 0.188 | 1.205 | 0.266 | 1.182 |
| 12:32893715 | T | G | 2/47/1813 | 0/39/1196 | 0.014 | 0.016 | 0.809 | 0.950 | 0.516 | 0.866 |
| 12:32894129 | T | C | 11/212/1644 | 7/138/1066 | 0.063 | 0.063 | 0.961 | 1.005 | 1.000 | 0.999 |
| 12:32894215 | G | C | 12/207/1656 | 7/130/1072 | 0.062 | 0.060 | 0.595 | 1.059 | 0.785 | 1.037 |
| 12:32894258 | T | C | 11/210/1650 | 6/138/1066 | 0.062 | 0.062 | 0.973 | 1.004 | 1.000 | 1.000 |
| 12:32894269 | C | G | 11/203/1651 | 6/138/1061 | 0.060 | 0.062 | 0.808 | 0.974 | 0.785 | 0.967 |
| 12:32894603 | A | G | 11/211/1634 | 6/143/1063 | 0.063 | 0.064 | 0.914 | 0.989 | 0.872 | 0.980 |
| 12:32894681 | C | T | 8/191/1657 | 4/121/1097 | 0.056 | 0.053 | 0.679 | 1.049 | 0.647 | 1.060 |
| 12:32894729 | T | C | 10/197/1633 | 5/135/1070 | 0.059 | 0.060 | 0.951 | 1.007 | 0.912 | 0.983 |
| 12:32894781 | T | C | 11/212/1644 | 5/141/1059 | 0.063 | 0.063 | 0.857 | 1.020 | 1.000 | 1.000 |
| 12:32896365 | C | G | 9/212/1624 | 8/145/1060 | 0.062 | 0.066 | 0.609 | 0.947 | 0.557 | 0.935 |
| 12:32896366 | T | A | 9/207/1633 | 8/144/1058 | 0.061 | 0.066 | 0.502 | 0.931 | 0.420 | 0.915 |
| 12:32896615 | G | C | 9/216/1647 | 7/135/1067 | 0.063 | 0.062 | 0.791 | 1.029 | 0.914 | 1.015 |
| 12:32897088 | T | A | 10/200/1649 | 6/141/1075 | 0.059 | 0.063 | 0.641 | 0.951 | 0.585 | 0.942 |
| 12:32897437 | C | T | 9/215/1651 | 6/143/1082 | 0.062 | 0.063 | 0.992 | 1.001 | 0.915 | 0.986 |
| 12:32897557 | T | G | 8/207/1629 | 8/145/1057 | 0.060 | 0.067 | 0.409 | 0.916 | 0.360 | 0.903 |
| 12:32897586 | A | G | 8/211/1631 | 7/149/1076 | 0.061 | 0.066 | 0.538 | 0.936 | 0.455 | 0.923 |
| 12:32898599 | T | C | 0/38/1813 | 0/33/1181 | 0.010 | 0.014 | 0.279 | 0.768 | 0.272 | 0.753 |
| 12:32899929 | G | C | 10/197/1626 | 8/130/1052 | 0.059 | 0.061 | 0.907 | 0.987 | 0.740 | 0.963 |
